# OTULIN confers cisplatin resistance in osteosarcoma by mediating GPX4 protein homeostasis to evade the mitochondrial apoptotic pathway

**DOI:** 10.1186/s13046-024-03249-8

**Published:** 2024-12-26

**Authors:** Zehang Zheng, Yunhao Zeng, Xing Bao, Chuang Huang, Fengjing Guo, Fei Xu, Zhengqiang Luo

**Affiliations:** Department of Orthopedics, Tongji Hospital, Tongji Medical College, Huazhong University of Science and Technology, Wuhan, China

**Keywords:** Osteosarcoma, Chemoresistance, OTULIN, GPX4, Apoptosis

## Abstract

**Background:**

Osteosarcoma (OS), the most prevalent primary malignant bone tumor in children and adolescents, arises from bone-forming mesenchymal cells. Despite advancements in surgical resection and neoadjuvant chemotherapy (cisplatin, doxorubicin, and methotrexate), chemotherapy resistance remains a significant challenge, leading to poor survival rates in patients with metastatic or recurrent OS.

**Methods:**

In this study, we focused on the role of OTULIN, a key linear deubiquitinating enzyme, in OS chemoresistance. In addition, mechanistic investigations were carried out to identify potential downstream targets of OTULIN involved in cisplatin resistance.

**Results:**

Our results demonstrated that OTULIN expression was significantly upregulated in OS tissues and cell lines following cisplatin treatment but not in response to doxorubicin or methotrexate. High OTULIN expression was associated with reduced survival in sarcoma patients. Furthermore, immunohistochemical analysis of prechemotherapy and postchemotherapy OS tissues revealed increased OTULIN expression in postchemotherapy samples. In vitro results demonstrated that OTULIN plays a critical role in mediating cisplatin resistance in OS. Mechanistically, GPX4 could be a downstream target of OTULIN, conferring cisplatin resistance to OS by blocking the mitochondrial apoptotic pathway but not ferroptosis. Specifically, OTULIN prevents the proteasomal degradation of GPX4 by reducing its ubiquitin level, thereby conferring resistance to cisplatin in OS cells.

**Conclusion:**

This study highlights the importance of OTULIN in OS chemoresistance and provides a promising approach for targeting the OTULIN-GPX4 axis to improve the prognosis of OS patients. Our findings offer new insights into the molecular mechanisms underlying OS chemoresistance and suggest potential therapeutic targets for future clinical interventions.

**Supplementary Information:**

The online version contains supplementary material available at 10.1186/s13046-024-03249-8.

## Introduction

Osteosarcoma (OS), the most common primary malignant bone tumor in children and adolescents, is derived from bone-forming mesenchymal cells [1]. Currently, the combination of surgical resection and neoadjuvant chemotherapy (cisplatin, doxorubicin, and methotrexate) has increased the 5-year overall survival (OS) rate from less than 20% to greater than 60% [2]. However, many patients with metastatic or recurrent OS have an overall survival rate of approximately 20% because chemotherapy resistance is currently still one of the causes of treatment failure in OS [3]. Several studies have shown that in OS patients who have a poor response to initial chemotherapy, either increasing the concentration of the first-line chemotherapeutic agent or adding other second-line chemotherapeutic agents has resulted in very limited efficacy [4]. Therefore, exploring new targets to reverse chemotherapy resistance to improve the prognosis of OS patients is important.

In recent years, OTU deubiquitinase with linear linkage specificity (OTULIN) has been identified as a key linear deubiquitinating enzyme involved in cell death and inflammation [5]. There is increasing evidence that abnormal linear ubiquitination is responsible for a spectrum of immune dysfunctions and inflammatory diseases [6]. It has been reported that the frequent overexpression of the linear chain-specific deubiquitinating enzyme OTULIN is associated with poor prognosis in glioblastoma patients [7]. However, the role of OTULIN in OS, especially in chemotherapy-resistant OS, has not been well investigated. Since OTULIN promotes cell survival in some cases [8], the relationship between OTULIN and chemoresistance in OS deserves attention.

In this study, we found that OTULIN expression was markedly upregulated following chemotherapy, with cisplatin being the primary agent responsible for this induction, as opposed to doxorubicin and methotrexate. High OTULIN expression inhibited cisplatin-induced apoptosis but not ferroptosis. Mechanistically, OTULIN maintains the stability of the GPX4 protein by regulating the ubiquitination level of GPX4, thereby conferring resistance to cisplatin in osteosarcoma cells. This study provides a promising approach for targeting the OTULIN-GPX4 axis for the treatment of OS chemoresistance.

## Method and materials

### Cell lines and culture

Six human OS cell lines (143B, KHOS, MG-63, SaOS-2, U2OS, HOS) were procured from Cyagen Biosciences (Guangzhou, China). The aforementioned cell lines were cultured in DMEM medium, supplemented with 10% fetal bovine serum and 100 U/ml penicillin/streptomycin solution (GIBCO, Gaithersburg, US), at 37 °C in a humidified incubator with 5% CO_2_ atmosphere.

### Human specimen collection

Tissue samples were collected from six patients with osteosarcoma diagnosed at Tongji Hospital, Tongji Medical College, Huazhong University of Science and Technology, China, between August 2020 and August 2023. The samples included biopsied tumor tissues before chemotherapy and excised tumor tissues after chemotherapy. The collected tumor tissues were embedded and sectioned for subsequent immunohistochemistry. All patients signed a written informed consent. The study was conducted in accordance with the principles set forth in the Declaration of Helsinki and was approved by the Ethics Committee of Tongji Hospital, Tongji Medical College, Huazhong University of Science and Technology (TJ-IRB202401005). Patients’ tumor information is provided in supplementary file 1.

### Bioinformatic analysis

In this study, the OS-related microarray dataset TCGA-SARC was obtained from TCGA (The Cancer Genome Atlas Program) dataset, and bioinformatics analyses were performed using these datasets. Survival analysis was performed using the R software package "survival" to investigate the correlation between the expression levels of key genes and prognosis, and to observe the differences in the expression of key genes in normal tissues and paracancerous tissues, and the above bioinformatic analyses were visualized using the R software.

### Reagents and antibodies

The commercial reagents and antibodies utilized in this study were described as follow: OTULIN (ab211328, Abcam), GPX4 (ab125066, Abcam), OTULIN (ab151117, Abcam), GPX4 (67,763–1-Ig, Proteintech), GAPDH (60,004–1-Ig, Proteintech), Ubiquitin (#43124S, CST), Ub-M1 (PA5-120,624, Thermo-Fisher Scientific), Ubiquitin linkage-specific K48 (ab140601, Abcam), Bcl-XL (10,783–1-AP, Proteintech), Bcl2 (12,789–1-AP, Proteintech), Bax (50,599–2-Ig, Proteintech), Caspase 3 (82,202–1-RR, Proteintech), Cleaved Caspase 3 (#9661, CST), Anti-DDDDK tag (ab205606, Abcam), Cisplatin (S1166, selleck), DMSO (HY–Y0320, MCE), Methotrexate (HY-14519, MCE), Doxorubicin hydrochloride (HY-15142, MCE), RSL3 (HY-100218A, MCE), PS341 (HY 10227, MCE), Erastin (S7242, Selleck), Fer-1 (S7243, Selleck), CHX (HY-12320, MCE), E64D (S7393, Selleck), N-Ethylmaleimide (S3692, Selleck), MG132 (S2619, Selleck), NAC (HY-B0215, MCE), SLC7A11 (A2413, ABclonal), ACSL4 (A20414, ABclonal), ALOX15 (A22908, ABclonal).

### Plasmids, Small interfering RNA (siRNA), lentivirus construction and CRISPR/Cas9 knockout cell lines

siRNA-GPX4 and siRNA-OTULIN were synthesized by TSINGKE (Beijing, China). Overexpression plasmid constructs for Flag-GPX4 and OTULIN were synthesized by GeneChem (Shanghai, China). Transfection was conducted using Lipofectamine 3000 Transfection Reagent (Thermo Fisher, UT, USA) in accordance with the manufacturer's instructions. The plasmids and siRNA were introduced into the cells for approximately 8 h. Thereafter, the cells were replaced with fresh medium and cultured for 48 h to allow for subsequent interventions.

OTULIN-interfering lentivirus prepared with the PLVX-GFP/PURO lentiviral plasmid was synthesized by TSINGKE (Beijing, China). After 72 h of infection, shRNA-OTULIN and shRNA-NC knockdown cells were screened with 10 μg/ml puromycin. GFP expression was detected using fluorescence microscopy to verify the transfection efficiency. Western blotting was utilized to verify the efficiency of lentivirus-mediated silencing of OTULIN in cells.

The CRISPR/Cas9 system was used to construct the OTULIN knockout osteosarcoma 143B cell line. Support for the CRISPR/Cas9 techniques was provided by AUCGT (Wuhan, China). Briefly, the human OTULIN genome sequence was first analyzed, two optimal sgRNA sequences were designed, and these sgRNA sequences were chemically synthesized. Subsequently, sgRNA and Cas9 protein complexes were electro-transfected into 143B cells for knockout (KO). Targeting was further confirmed by Sanger sequencing, and gene expression of the control and experimental KO monoclonal cell lines was detected by qPCR. Finally, the KO monoclonal cell lines were progressively expanded in culture.

The sequences of the relevant siRNA and sgRNA were provided in supplementary file 1.

### RNA extraction and RT-qPCR

RNA was extracted and purified from osteosarcoma cells using an RNA extraction kit (Omega Biotek, R6834-01, USA). The integrity, quantity, and purity of the obtained RNA samples were determined by a microplate reader (Thermo Fisher Scientific, Vantaa, Finland). Qualified RNA samples are required to have an A260/A280 ratio within the range of 1.8 to 2.0. Subsequently, the extracted RNA was reverse transcribed into cDNA using Hifair® III 1st Strand cDNA Synthesis SuperMix (YEASEN, 11141ES60, China), and the extracted RNA was subjected to RT-qPCR using Hieff® qPCR SYBR Green Master Mix (YEASEN, 11201ES03, China). Sequences of the primers were provided in supplementary file 1.

### Immunoprecipitation (IP)

Lysis was performed using NP-40 lysis buffer (AR0107, Boster, China) enriched with 1% protease inhibitor cocktail, 5 mM NaF, 1 mM Na3VO4, 10 mM β-glycerophosphate and 50 mM N-ethylmaleimide. The resulting lysate was incubated with the antibody for 2 h at 4 ℃, and then the target immune complex was efficiently captured by Protein A/G magnetic beads (HY-K0202, MCE, USA) overnight at 4 ℃. After three rigorous washes with NP-40 lysis buffer, the immune complexes were released from the magnetic beads by heating to 95 °C for 10 min using 2 × SDS-PAGE sample buffer.

During denaturing immunoprecipitation, cells were first lysed in a strong 1% SDS lysis buffer and then heated at 100 °C for 10 min. After a tenfold dilution in NP-40 Lysis Buffer, the lysates are immunoprecipitated using the appropriate antibodies and protein A/G beads.

### Western blot

The cells were washed three times with PBS at 4 °C, followed by lysis on ice for 30 min with RIPA lysis buffer (AR0102, Boster, China) containing a protease inhibitor cocktail. The resulting protein supernatants were subjected to 12,000 g centrifugation for 30 min, after which the protein concentration was determined using the BCA assay kit (AR0146, Boster, China). Equal masses of protein were subjected to SDS-PAGE gel electrophoresis and transferred to PVDF membranes (Millipore, USA). Following a 1-h incubation with 5% skimmed milk powder at room temperature, the membrane was treated with primary antibody overnight at 4 °C. Subsequently, it was incubated with secondary antibody for 1 h at room temperature. The protein bands were visualized using the ChemiDoc™ XRS^+^ system (Bio-Rad Laboratories, CA, USA). The density of each protein band was quantified using ImageJ software (v.1.51, USA). Each set of protein bands had three biological replicates.

### Immunohistochemistry (IHC)

Briefly, the tissue sections were initially deparaffinized and antigenically repaired, followed by incubation with the specified antibodies at 4 °C overnight. On the following day, the sections were stained with BCIP/NBT (AR1023, Boster, China) following an incubation period of 1 hour at room temperature with biotinylated goat anti-rabbit IgG secondary antibody. Images were obtained using a fluorescence microscope (Evos flauto, Life Technologies, USA). The number of positive cells per field of view in each group was counted at 400× magnification.

### Transmission electron microscopy (TEM) observation

In brief, PBS-flushed osteosarcoma cells were fixed with electron microscopy fixative (G1102, Servicebio, China), followed by dehydration of the samples with different concentrations of alcohol and acetone. Subsequently, the samples were rinsed with propylene oxide and impregnated with epoxy resin. The ultrathin sections were then stained with 1% uranyl acetate and 0.1% lead citrate. Scanning was performed using the Hitachi TEM system.

### Cell counting kit 8 (CCK 8) assay

Osteosarcoma cells were cultured into 96-well plates at a density of 5,000 cells per well, and each set of experiments was repeated in five wells. The viability of the cells was determined using the Cell Counting Kit-8 (HY-K0301, MCE). Following the removal of treated cells from the 96-well plates, 100 µl of 10% CCK-8 solution was added and the plates were incubated at 37 °C for one hour under light protection. Absorbance at 450 nm was then measured with a microplate reader (Thermo Fisher Scientific, Vantaa, Finland). Cell survival was normalized to that of control cells treated with vector only.

### Colony formation assay

Cell proliferation ability was measured by plate colony formation assay. Briefly, 500 cells were added to each well of a 6-well plate and incubated for 2 weeks until a colony was obviously formed; the medium was regularly changed. Next, the plate was gently washed and stained with 0.1% crystal violet, and the number of colonies was counted.

### Cell migration and invasion assay

A wound-healing assay was used to test cell migration ability. Briefly, cells in the exponential phase of growth were harvested and seeded in a 6-well plate. After the cell reached 90% confluence, a line was drawn using a marker on the bottom of the dish, after which a sterile 100 μl pipet tip was used to scratch three separate wounds through the cells, moving perpendicular to the line. Next, the cells were gently rinsed twice with PBS to remove floating cells. Images of the scratches were taken using an inverted microscope (Evos flauto, Life technologies, USA) at × 10 magnification at 0 and 24 h of incubation.

The cell invasion assay was performed with Corning Matrigel Basement Membrane Matrix (Corning Inc, Corning, NY, USA). The Matrigel was thawed, diluted, and added to the growth surface of the transwell chamber (Labselect, Hangzhou, China) followed by resting at 37˚C for 1 h. The 143B cells and U2OS cells in the logarithmic growth phase were collected to perform trypsin digestion and serum-free medium resuspension. After that, we added 100 μL cell suspension to the upper chamber and added 600 μL 20% serum medium in the lower chamber. After 24 h incubation, we removed the culture and washed the transwell chamber with PBS. The polyethylene terephthalate (PET) membrane was immersed in 70% methanol to fix the cells, which were subsequently stained by crystal violet solution. Ultimately, we used the inverted fluorescence microscope (Evos flauto, Life technologies, USA) to obtain the final result.

### Total, mitochondrial and lipid reactive oxygen species (ROS) assay

For total ROS assay, cells were rinsed with PBS and treated with 10 μM DCFH-DA (HY-D0940, MCE) for 30 min at 37 °C in the dark. After incubation, cells were rinsed 3 times with PBS, observed and recorded under a fluorescence microscope (Evos flauto, Life Technologies, USA).

For mitochondrial ROS assay, cells were trypsinized and resuspended in a culture medium. The cells were rinsed with PBS and then treated with 10 μM MitoSOX Red (HY-D1055, MCE) and kept at 37 °C for 30 min away from light. After incubation, the cells were washed three times with PBS. Fluorescence (510/580 nm) intensity was simultaneously captured using a flow cytometer (Neon-1026 M, China) and analyzed using FlowJo V10 software.

For lipid ROS, cells were treated with 10 μM C11-BODIPY 581/591 Lipid Peroxidation Sensor (D3861, ThermoFisher Scientific) for 30 min at 37 °C under dark conditions after rinsing in PBS. After incubation, the fluorescence intensity in green (484/510 nm) was captured using a flow cytometer (Neon-1026 M, China) and analyzed using FlowJo V10 software.

### Apoptosis detection and JC-1 assay

Apoptosis rate was assayed using Annexin V-FITC/PI staining kit (HY-K1073, MCE). Cells were resuspended after trypsin digestion. Then 195 μL of Annexin V-FITC conjugate was added and mixed, and 5 μL of Annexin V-FITC and 10 μL of PI staining solution was added and gently mixed. Cells were incubated at room temperature for 20 min, protected from light. Subsequently, the apoptosis rate was analyzed using a flow cytometer (Neon-1026 M, China). For JC-1 assay, cells were rinsed with PBS and treated with 2 μM JC-1 (HY-K0601, MCE) for 20 min at 37 °C in the dark. After incubation, cells were rinsed 3 times with PBS, observed and recorded under a fluorescence microscope (Evos flauto, Life Technologies, USA).

### Animal experiment

The experimental procedures involving animals were conducted in strict accordance with the U.K.'s Animals (Scientific Procedures) Act of 1986, as well as the European guidelines (2010/63/EU), and received approval from the Tongji Hospital Animal Welfare Ethics Committee at Tongji Medical College, Huazhong University of Science and Technology, Wuhan, China (TJH-202306007). 24 nude mice were bred in a specific pathogen-free (SPF) animal laboratory. The OS model of nude mice was constructed using the Construction of the cell line derived tumor xenograft (CDTX) model. 143B-shNC/143B-shOTULIN cells were mixed with matrigel and then injected subcutaneously into the right dorsal subcutis of 24 (6 per group) 4-week-old nude mice (1 × 10^7^ cells per mouse). Following injection, PBS and Cisplatin at a dosage of 3 mg/kg were administered via the intraperitoneal route, commencing from day 4 onwards, with subsequent administrations being repeated at an interval of three days. Throughout the experiment, the volume of the tumors was measured using vernier calipers, with 5 measurements every 4 days to ensure accuracy. Ultimately, on day 16, the mice underwent euthanasia, and the excised tumors were subsequently subjected to a weighing process.

### Immunofluorescence (IF)

Briefly, cells were treated, fixed with 4% paraformaldehyde for 30 min, permeabilized with 0.1% Triton X-100 (Sigma-Aldrich, T8787) for 5 min, blocked with 5% BSA in TBST for 1 h, incubated with primary and secondary antibodies, stained with DAPI (AR1177, Boster, China), and visualized under a fluorescence microscope (OLYMPUS FV31S-SW, Japan).

### Statistical analysis

Quantitative data were presented as means ± S.D.s. (standard deviation). Statistical analyses were conducted utilizing GraphPad Prism 9.0 software (GraphPad Software, USA). To assess differences in quantitative data between two groups, Student's two-tailed t-test was employed. For comparisons involving more than two groups, a one-way analysis of variance (ANOVA) was performed, followed by post hoc multiple comparisons. For nonparametric data, the Mann–Whitney U test was used for two comparisons, and the Kruskal–Wallis test was applied for multiple comparisons. In all instances, statistical significance was defined as *P* < 0.05, with * indicating *P* < 0.05, ** indicating *P* < 0.01, and NS indicating not significant.

## Results

### Cisplatin, but not doxorubicin or methotrexate, upregulated OTULIN expression in osteosarcoma

**Fig. 1:**
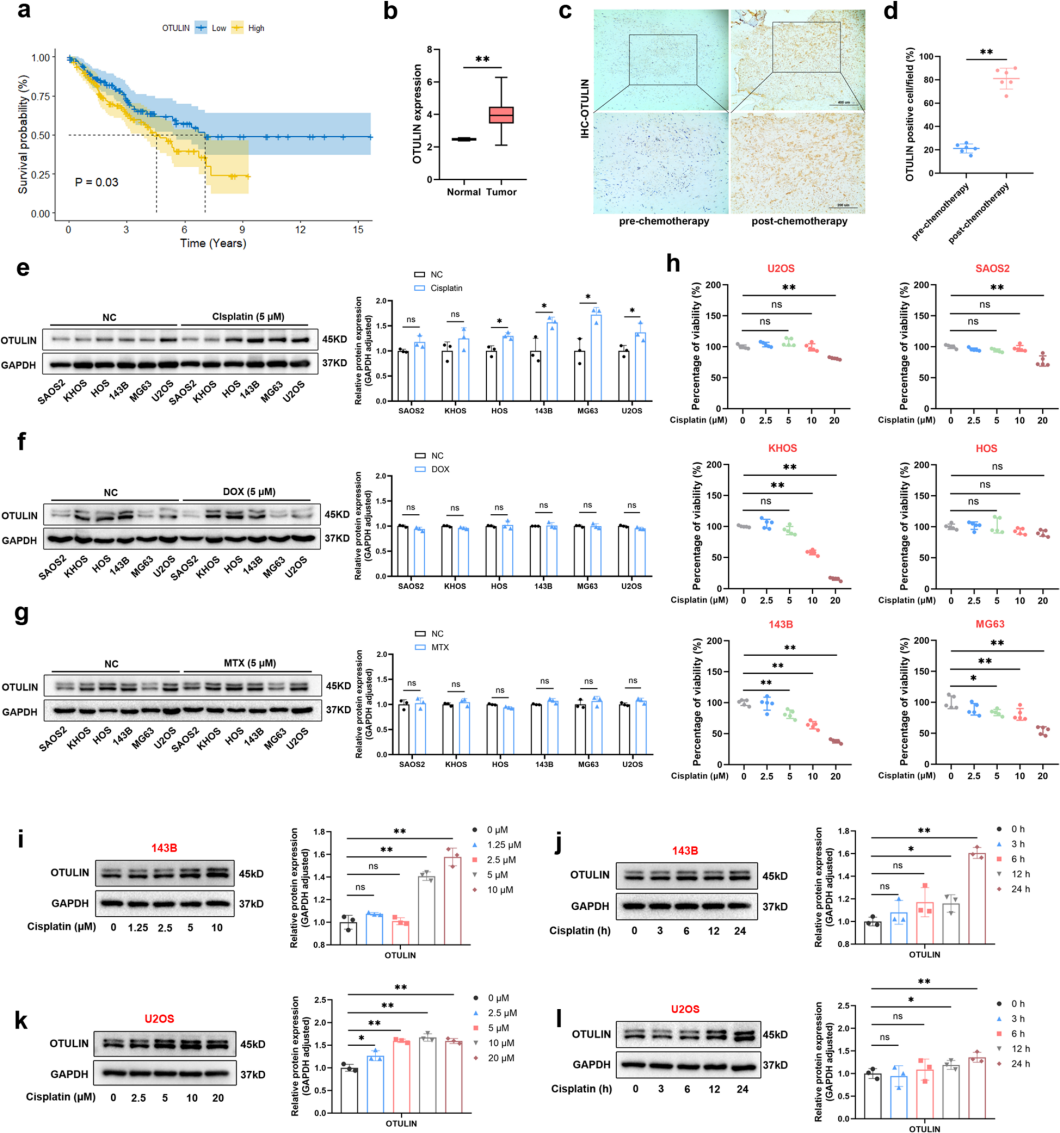
Cisplatin, but not doxorubicin or methotrexate, upregulated OTULIN expression in osteosarcoma. **a** Survival analysis of sarcoma patients in the TCGA database and OTULIN expression. **b** Comparison of differences in OTULIN expression in paraneoplastic and tumor tissues from sarcoma patients in the TCGA database.** c**,** d** Representative IHC images of OTULIN and quantitative analysis of OTULIN-positive cells (*n* = 6) (scale bars: 200 μm and 400 μm).** e-g** Western blot results and semiquantitative analysis of band density in different OS cell lines treated with cisplatin (5 μM), doxorubicin (DOX) (5 μM) or methotrexate (MTX) (5 μM), as indicated, for 24 h (*n* = 3). **h** CCK-8 assays were performed to assess the viability of different OS cell lines treated with increasing concentrations of cisplatin (0, 2.5, 5, 10, or 20 μM) for 24 h (*n* = 5). **i-l** Western blot results and semiquantitative analysis of the band density of 143B and U2OS cells treated with different concentrations of cisplatin for 24 h or with different durations of treatment with 10 μM cisplatin. The values are expressed as means ± S.D.s. *P < 0.05, **P < 0.01 vs. the corresponding control

To explore the effect of OTULIN expression on the survival of patients with tumors, we extracted data from the TCGA database on OTULIN associated with sarcoma (data on osteosarcoma were not used because there is more abundant data on sarcoma than osteosarcoma) and found that high OTULIN expression significantly reduced the survival of patients with sarcoma (Fig. 1a). In addition, a comparison of the mRNA expression data of paraneoplastic and tumor tissues revealed that the expression of OTULIN in tumors was significantly greater than that in paraneoplastic tissues (Fig. 1b). Next, we collected tumor specimens from prechemotherapy and postchemotherapy osteosarcoma patients admitted to our hospital and found via immunohistochemistry that OTULIN expression was higher in postchemotherapy patients (*n* = 6) than in prechemotherapy patients (*n* = 6) with OS (Fig. 1c, d). These results suggested that our OS chemotherapy regimen induced an increase in OTULIN expression. To assess OTULIN expression in vitro, we treated six osteosarcoma cell lines (143B, U2OS, SAOS-2, HOS, KHOS and MG63) with different chemotherapeutic agents (cisplatin, doxorubicin, or methotrexate) for 24 h. The results revealed that only cisplatin significantly increased the protein level of OTULIN, whereas doxorubicin or methotrexate did not (Fig. 1e–g). Additionally, the CCK-8 results revealed that these six osteosarcoma cell lines differed in the degree of resistance to cisplatin (Fig. 1h). Therefore, we selected the less resistant 143B cell line and the more resistant U2OS cell line to investigate cisplatin-induced OTULIN expression and found that cisplatin induced an increase in OTULIN expression in a concentration-dependent and time-dependent manner (Fig. 1i-j). On the basis of these results, we hypothesized that cisplatin-stimulated high OTULIN expression might promote chemoresistance in OS and reduce patient survival.

### OTULIN knockdown markedly decreased cisplatin resistance in osteosarcoma

**Fig. 2:**
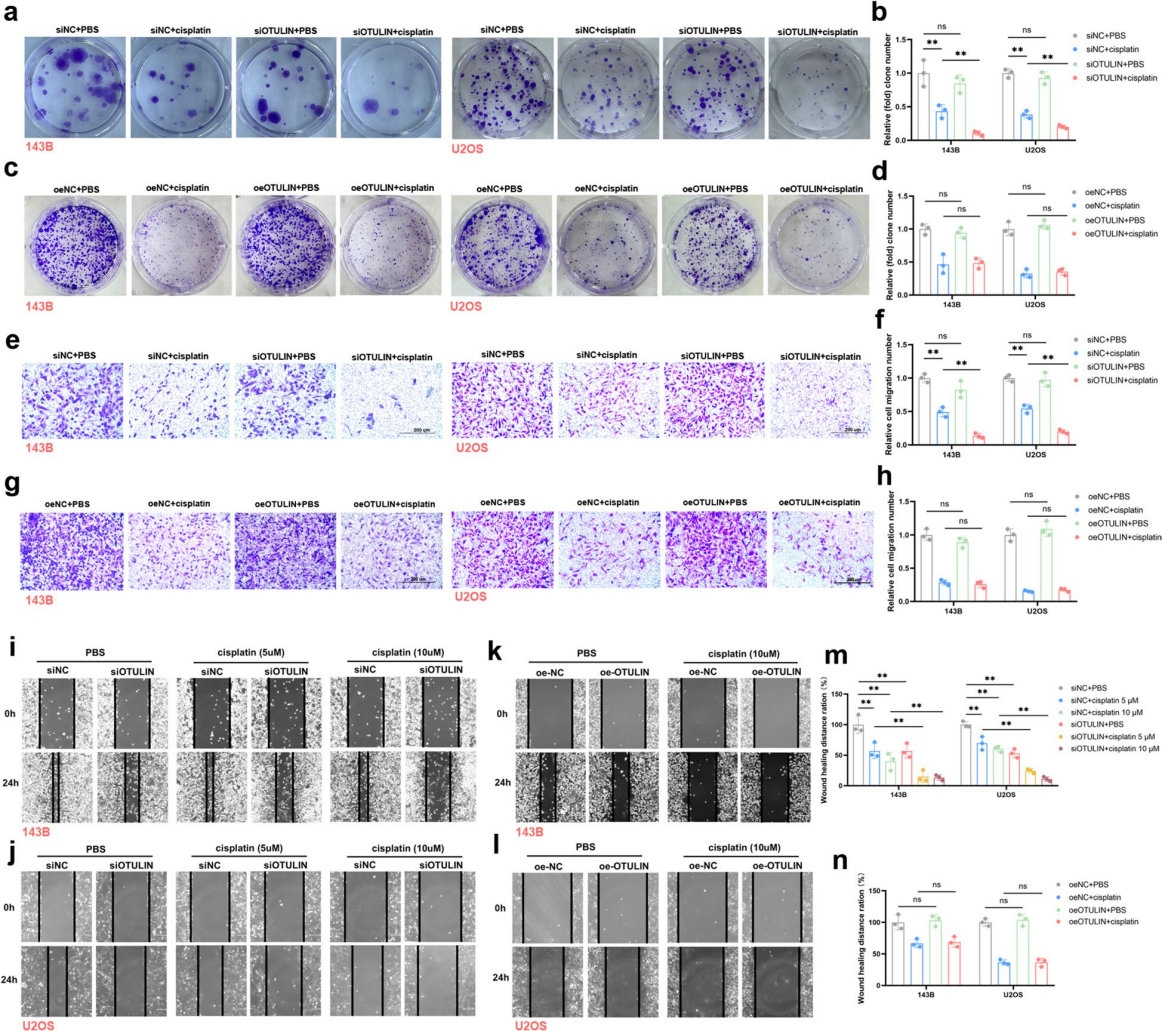
OTULIN knockdown markedly decreased cisplatin resistance in osteosarcoma. **a**-**d** Representative images and relative quantitative analysis of colony formation experiments; osteosarcoma cells were transfected with siRNA or plasmid for 48 h and then treated with cisplatin (5 μM) for 2 weeks.** e-f** Representative images and relative quantitative analysis of invasion assays; osteosarcoma cells were transfected with siRNA or plasmid for 48 h and then treated with cisplatin (143B, 5 μM; U2OS, 10 μM) for 24 h. **i**-**n** Representative images and relative quantitative analysis of wound healing assays; osteosarcoma cells were transfected with siRNA or plasmid for 48 h and then treated with the indicated concentrations of cisplatin for 24 h. The values are expressed as means ± S.D.s. *P < 0.05, **P < 0.01 vs. the corresponding control

**Fig. 3:**
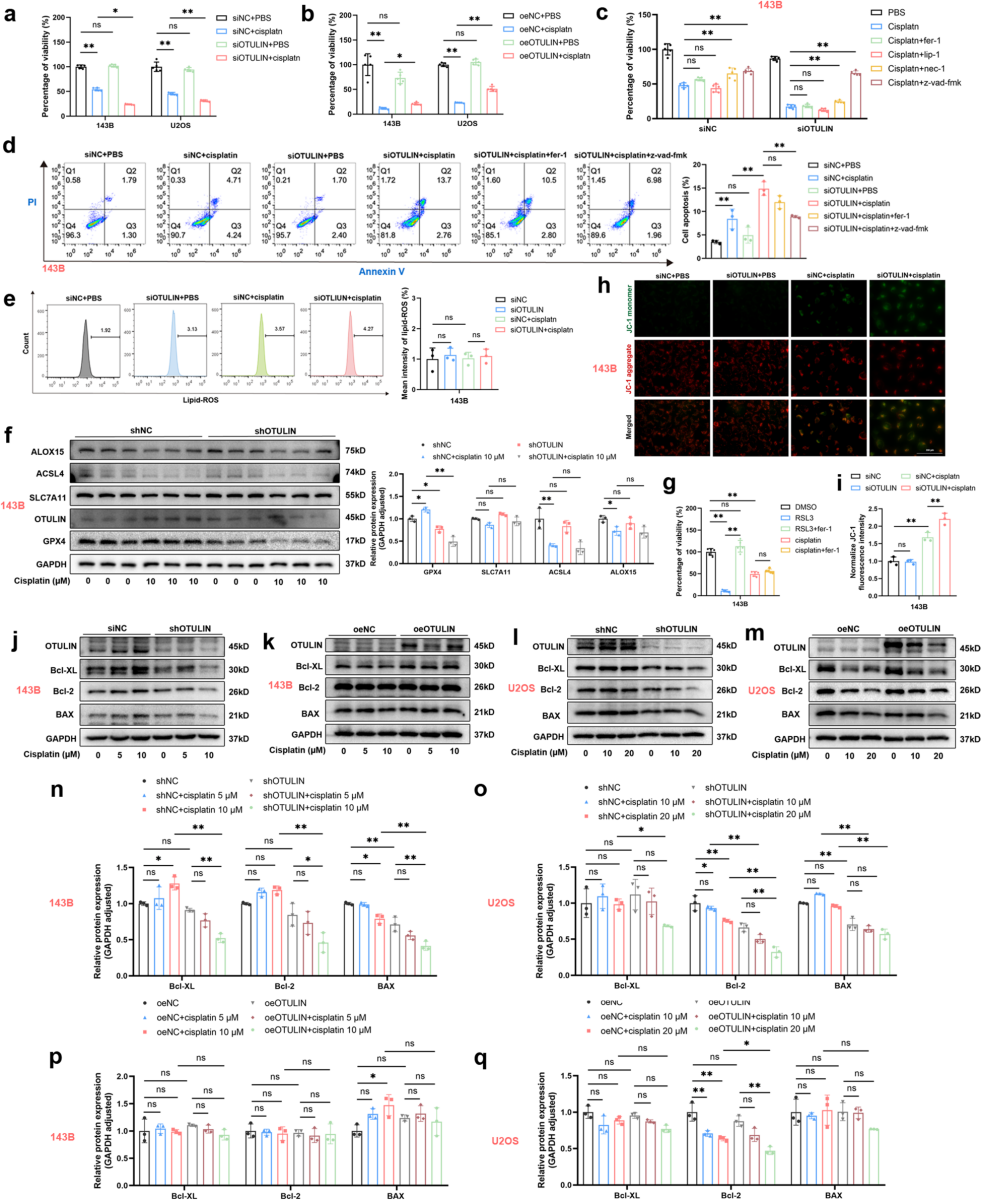
OTULIN knockdown promoted cisplatin-induced apoptosis but not ferroptosis in osteosarcoma cells. OS cells were transfected with siRNA or plasmids for 48 h for subsequent interventions. **a**,** b** Transfected OS cells were treated with cisplatin (10 μM for 143B cells and 20 μM for U2OS cells) for 24 h, and cell viability was assessed using CCK8 assays. **c** Transfected 143B cells were pretreated with 10 μM inhibitor (fer-1, lip-1, nec-1, or Z-VAD-FMK) for 1 h and then treated with cisplatin (10 μM) for 24 h. Cell viability was assayed by CCK8 assays. **d** Transfected 143B cells were pretreated with 10 μM fer-1 or Z-VAD-FMK for 1 h and then treated with cisplatin (10 μM) for 24 h; apoptosis levels were assessed by flow cytometry (*n* = 3). **e** Lipid-ROS levels in transfected 143B cells treated with cisplatin (10 μM) for 24 h were assessed via flow cytometry. Statistical analysis of fluorescence intensity was performed using FlowJo V10 software (*n* = 3). **f** Western blot results and semiquantitative analysis of the band density for 143B cells transfected with the shRNA-OTULIN lentivirus. Cells were treated with cisplatin (10 μM) for 24 h. **g** 143B cells were pretreated with 10 μM fer-1 or DMSO and then treated with cisplatin (10 μM) or RSL3 (0.5 μM) for 24 h. Cell viability was assessed using CCK8 assays. **h**,** i** Representative images of JC-1 staining (scale bar: 200 μm) and statistical analysis of fluorescence intensity (*n* = 3). Transfected 143B cells were treated with cisplatin (10 μM) for 24 h. **j-q** Western blot results and semiquantitative analysis of the band density for 143B or U2OS cells transfected with shRNA-OTULIN lentivirus or an OTULIN overexpression plasmid. Cells were treated with cisplatin for 24 h (5/10 μM for 143B cells and 10/20 μM for U2OS cells). The values are expressed as means ± S.D.s. *P < 0.05, **P < 0.01 vs. the corresponding control

The aforementioned hypothesis was tested in vitro. Small interfering RNAs were used to knock down OTULIN expression, and the interference efficiency is shown in Supplementary File 1 (sFig. 1). The results of the cloning experiments demonstrated that compared with that in the NC, OTULIN knockdown had no significant effect on the proliferation of osteosarcoma cells. However, in the context of cisplatin intervention, OTULIN knockdown markedly enhanced the inhibitory effect of cisplatin on proliferation (Fig. 2a, b). The results of the invasion assay also demonstrated that OTULIN knockdown alone had no effect on the invasive capacity of osteosarcoma cells. However, OTULIN knockdown significantly enhanced the inhibitory effect of cisplatin on the invasive capacity of osteosarcoma cells (Fig. 2e, f). In contrast, the results of the wound healing assay demonstrated that compared with the control, OTULIN knockdown alone impeded the migratory capacity of osteosarcoma cells. Furthermore, OTULIN knockdown enhanced the inhibitory effect of cisplatin on osteosarcoma cell migration (Fig. 2i, j, m). Notably, OTULIN overexpression had no significant effect on the proliferative, invasive, or migratory capacities of osteosarcoma cells with or without cisplatin administration (Fig. 2c, d, g, h, k, l, n). This may be attributed to the fact that cisplatin-induced high OTULIN expression was already present. Furthermore, the CCK8 results demonstrated that OTULIN knockdown enhanced cisplatin-induced cell death, whereas OTULIN overexpression impeded the cisplatin-induced reduction in cell viability (Fig. 3a, b). These findings collectively indicate that OTULIN deficiency decreases cisplatin resistance in osteosarcoma cells.

### OTULIN knockdown promotes cisplatin-induced apoptosis but not ferroptosis in osteosarcoma

Apoptosis is known to be involved in cisplatin-induced cell death, and ferroptosis has also been reported to be involved in cisplatin-induced cell death [9, 10]; therefore, to investigate which cell death type OTULIN specifically modulates to affect cisplatin-induced cell death, we administered Z-VAD-FMK (a pancaspase inhibitor), necrostatin-1 (nec-1, a RIPK1 inhibitor), ferrostatin-1 and liproxstatin-1 (fer-1 and lip-1, ferroptosis inhibitors) 1 h before cisplatin administration. The results revealed that Z-VAD-FMK, but not fer-1 or lip-1, significantly reversed, and nec-1 partially reversed, cisplatin-induced cell death exacerbated by OTULIN knockdown (Fig. 3c). Moreover, the flow cytometry results revealed that the apoptosis rate of OTULIN-knockdown osteosarcoma cells after cisplatin intervention was reduced by Z-VAD-FMK, whereas that of fer-1-treated osteosarcoma cells was not (Fig. 3d).

Elevated levels of membrane lipid peroxidation is a critical factor in the mechanism of ferroptosis [11]. To exclude a role for ferroptosis, we examined lipid peroxidation levels in osteosarcoma cells. Flow cytometry results revealed that cisplatin, with or without OTULIN knockdown, did not significantly increase lipid peroxidation levels (Fig. 3e). Concurrently, although the expression of GPX4, a principal anti-lipid peroxidation protein, was shown to be diminished in our findings, modifications of other pivotal proteins with respect to ferroptosis, including SLC7A11, ACSL4 and ALOX15, did not mirror the occurrence of ferroptosis. (Fig. 3f). Additionally, fer-1 reversed RSL3-inhibited cell viability but not cisplatin-inhibited cell viability (Fig. 3g).

To further clarify the role of apoptosis, we subsequently examined alterations in the mitochondrial membrane potential via JC-1 (mitochondrial membrane potential) assays. The results indicated that OTULIN knockdown significantly exacerbated the cisplatin-induced alterations in the mitochondrial membrane potential (Fig. 3h, i). Next, to facilitate subsequent experiments, we used a lentiviral plasmid to construct 143B and U2OS OTULIN-knockdown cell lines. BCL-2 and BCL-XL are considered pivotal biomarkers of the mitochondrial apoptotic pathway [12]. Western blot results demonstrated that OTULIN knockdown markedly enhanced the cisplatin-induced reduction in the expression of the antiapoptotic cellular markers Bcl-2 and Bcl-XL. Conversely, OTULIN overexpression partially reversed the cisplatin-induced decrease in Bcl-2 and Bcl-XL expression (Fig. 3j-q). Taken together, our results indicate that OTULIN knockdown facilitates mitochondrial pathway apoptosis, but not ferroptosis, in cisplatin-treated osteosarcoma cells.

### GPX4 interacts with OTULIN and reverses cisplatin-induced cell death promoted by OTULIN knockdown

**Fig. 4:**
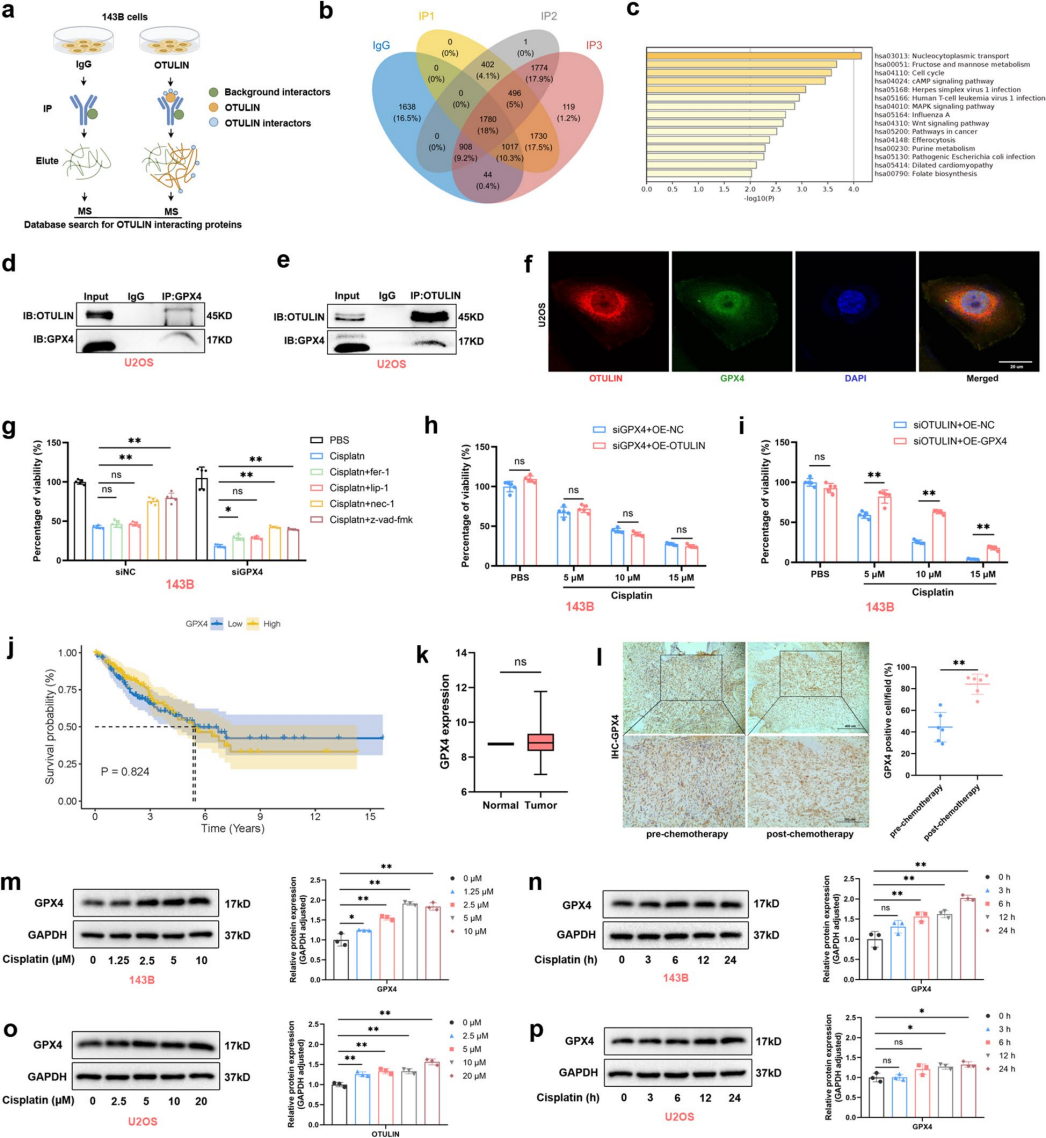
GPX4 interacted with OTULIN and reversed the cisplatin-induced cell death promoted by OTULIN knockdown. **a** Schematic representation of the LC‒MS procedure. Three replications were performed for the IP-OTULIN group. **b** Venn diagram showing the LC‒MS results for 496 interacting proteins. **c** KEGG pathway enrichment analysis of 496 proteins that interact with OTULIN. **d-e** Cell lysates of untreated U2OS cells were immunoprecipitated with the indicated antibodies and analyzed by Western blotting. **f** Representative images of immunofluorescence staining for the colocalization of GPX4 and OTULIN in U2OS cells (scale bar: 20 μm).** g** 143B cells transfected with siRNA for 48 h were pretreated with 10 μM inhibitor (fer-1, lip-1, nec-1, or Z-VAD-FMK) for 1 h and then treated with cisplatin (10 μM) for 24 h. Cell viability was assayed by CCK8 assays. **h**,** i** 143B cells cotransfected with the indicated siRNA and overexpression plasmids for 48 h were treated with PBS or cisplatin (5, 10 or 15 μM) for 24 h. **j** Survival analysis of sarcoma patients in the TCGA database and GPX4 expression. **k** Comparison of differences in GPX4 expression in paraneoplastic and tumor tissues from sarcoma patients in the TCGA database. **l** Representative IHC images of GPX4 and quantitative analysis of GPX4-positive cells (*n* = 6) (scale bars: 200 μm and 400 μm). **m-p** Western blot results and semiquantitative analysis of the band density for 143B and U2OS cells treated with different concentrations of cisplatin for 24 h or with different durations of treatment with 10 μM cisplatin. The values are expressed as means ± S.D.s. *P < 0.05, **P < 0.01 vs. the corresponding control

OTULIN, a specific linear deubiquitinating enzyme, primarily regulates the linear ubiquitin modification of target proteins, thereby affecting their function [6]. To elucidate the mechanism by which OTULIN promotes resistance to apoptosis in osteosarcoma cells, we conducted immunoprecipitation (IP) analysis of 143B cells utilizing an anti-OTULIN-specific antibody (an anti-IgG antibody was used as a control). The samples were analyzed for potential interacting proteins via liquid chromatography‒mass spectrometry (LC‒MS) (Fig. 4a). As shown in a Venn diagram, compared with the anti-IgG antibody, the anti-OTULIN antibody yielded 496 proteins, including the anti-lipid peroxidation protein GPX4 (see Supplementary File 2, Sheet 2) (Fig. 4b). Kyoto Encyclopedia of the Genome (KEGG) pathway analysis further revealed that cancer pathways were highly enriched (Fig. 4c).

Previous studies have indicated that GPX4, an antiapoptotic factor, primarily utilizes GSH as a cofactor to resist lipid peroxidation, thereby protecting membrane integrity [13]. Next, we verified the interaction of GPX4 with OTULIN using IP experiments and found that OTULIN could interact with GPX4 (Fig. 4d-e). We then investigated the mutual localization of GPX4 and OTULIN by immunofluorescence colocalization in U2OS cells under normal conditions and found that OTULIN and GPX4 were mainly localized in the cytoplasm; in the cytoplasm, the fluorescence of GPX4 and OTULIN overlapped (Fig. 4f). Previous studies reported that GPX4 might act as an inhibitory molecule of the mitochondrial apoptotic pathway; therefore, we hypothesized that GPX4 might be a downstream target protein of OTULIN.

To test this hypothesis, we explored whether GPX4 knockdown exacerbates cisplatin-induced cell death, and CCK8 assay results indicated both Z-VAD-FMK and nec-1 significantly reversed and fer-1 partially reversed, but lip-1 did not, the cisplatin-induced cell death exacerbated by GPX4 deficiency (Fig. 4g). These results suggest that GPX4 deficiency exacerbated a type of cisplatin-induced cell death more strongly in favor of apoptosis. Next, we found that cisplatin-induced cell death exacerbated by OTULIN deficiency could be reversed by GPX4 overexpression, and interestingly, in contrast, cisplatin-induced cell death exacerbated by GPX4 deficiency could not be reversed by OTULIN overexpression (Fig. 4h, i). These data suggest that GPX4 could be a potential target of OTULIN for resistance to cisplatin-induced apoptosis.

### GPX4 expression is upregulated following chemotherapy, and OTULIN is required for high GPX4 expression in response to cisplatin

To explore the effect of GPX4 expression on the survival of patients with tumors, we extracted data from the TCGA database on GPX4 associated with sarcoma and found that high GPX4 expression had no significant effect on the survival of patients with sarcoma and that the difference between GPX4 expression in tumors and that in paraneoplastic tissues was not significant (Fig. 4j, k). However, in the tumor specimens collected at our hospital, immunohistochemistry revealed that GPX4 expression was higher in postchemotherapy patients (*n* = 6) than in prechemotherapy patients (*n* = 6) with OS (Fig. 4l). These results suggest that GPX4 expression was upregulated after chemotherapy. Therefore, we subsequently investigated these findings in vitro and found that cisplatin induced an increase in GPX4 expression in a concentration- and time-dependent manner (Fig. 4m–n).

**Fig. 5:**
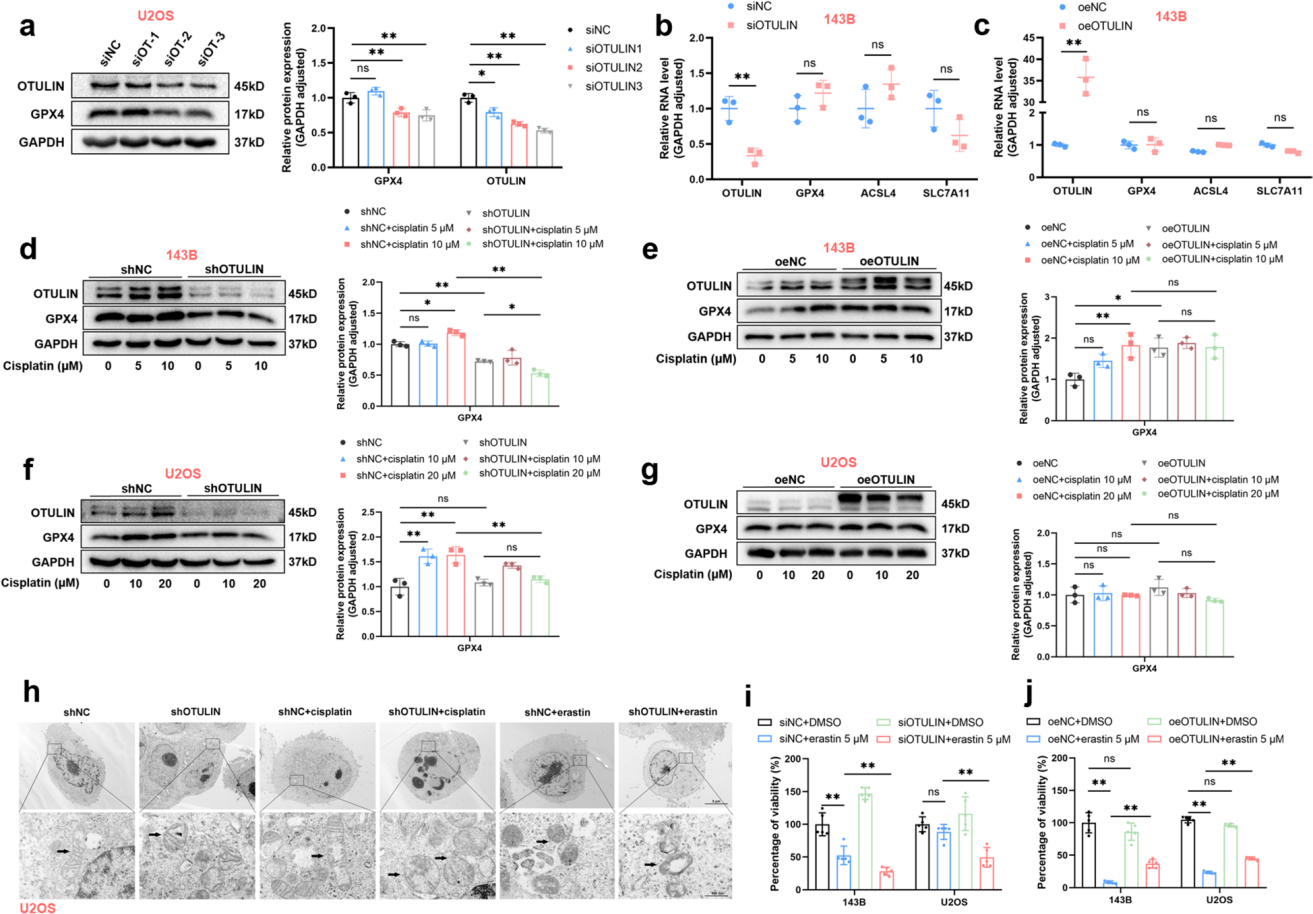
OTULIN was required for high GPX4 expression in response to cisplatin. **a** Western blot results and semiquantitative analysis of the band density for U2OS cells transfected with siRNA-OTULIN for 48 h. **b-c** RT‒qPCR results for 143B cells transfected with OTULIN siRNA or an OTULIN-overexpression plasmid for 48 h. **d-g** Western blot results and semiquantitative analysis of the band density for 143B or U2OS cells transfected with OTULIN shRNA lentivirus or an OTULIN overexpression plasmid. Cells were treated with cisplatin for 24 h (5/10 μM for 143B cells and 10/20 μM for U2OS cells). **h** Representative TEM images of mitochondria in U2OS cells; the black arrows indicate mitochondria (scale bars: 5 μm and 500 nm). **i**,** j** OS cells transfected with the indicated siRNA or plasmid for 48 h were treated with erastin (5 μM) for 24 h. Cell viability was assessed using CCK8 assays. The values are expressed as means ± S.D.s. *P < 0.05, **P < 0.01 vs. the corresponding control

Next, we hypothesized that the high protein expression of GPX4 induced by cisplatin is dependent on OTULIN. First, we found that OTULIN knockdown significantly inhibited GPX4 expression (Fig. 5a). qPCR results revealed that neither the knockdown nor the overexpression of OTULIN affected the transcription level of GPX4 (Fig. 5b, c). Furthermore, OTULIN knockdown significantly inhibited the cisplatin-induced increase in GPX4 protein expression, whereas OTULIN overexpression had no significant effect. (Fig. 5d-g). In addition, to further demonstrate that OTULIN promotes cisplatin-induced apoptosis rather than ferroptosis, we observed cellular and mitochondrial morphology via electron microscopy. The electron microscopy results revealed that OTULIN deficiency promoted cisplatin-induced death in osteosarcoma cells, which exhibited a morphology closer to that of apoptotic cells and aggravated mitochondrial damage, including mitochondrial swelling and membrane rupture (Fig. 5h). Interestingly, OTULIN knockdown also promoted changes in cellular and mitochondrial ferroptosis mediated by erastin, a ferroptosis inducer, including mitochondrial wrinkling, mitochondrial membrane thickening, and a reduction in the number of mitochondrial cristae (Fig. 5h). The CCK8 results also revealed that OTULIN knockdown significantly reduced the viability of osteosarcoma cells treated with erastin, whereas OTULIN overexpression increased the survival of osteosarcoma cells after erastin treatment. (Fig. 5i, j). In summary, these results indicate that OTULIN is required for high GPX4 protein expression in response to cisplatin.

### The OTULIN-GPX4 axis confers resistance to cisplatin in osteosarcoma by blocking the mitochondrial apoptotic pathway

**Fig. 6:**
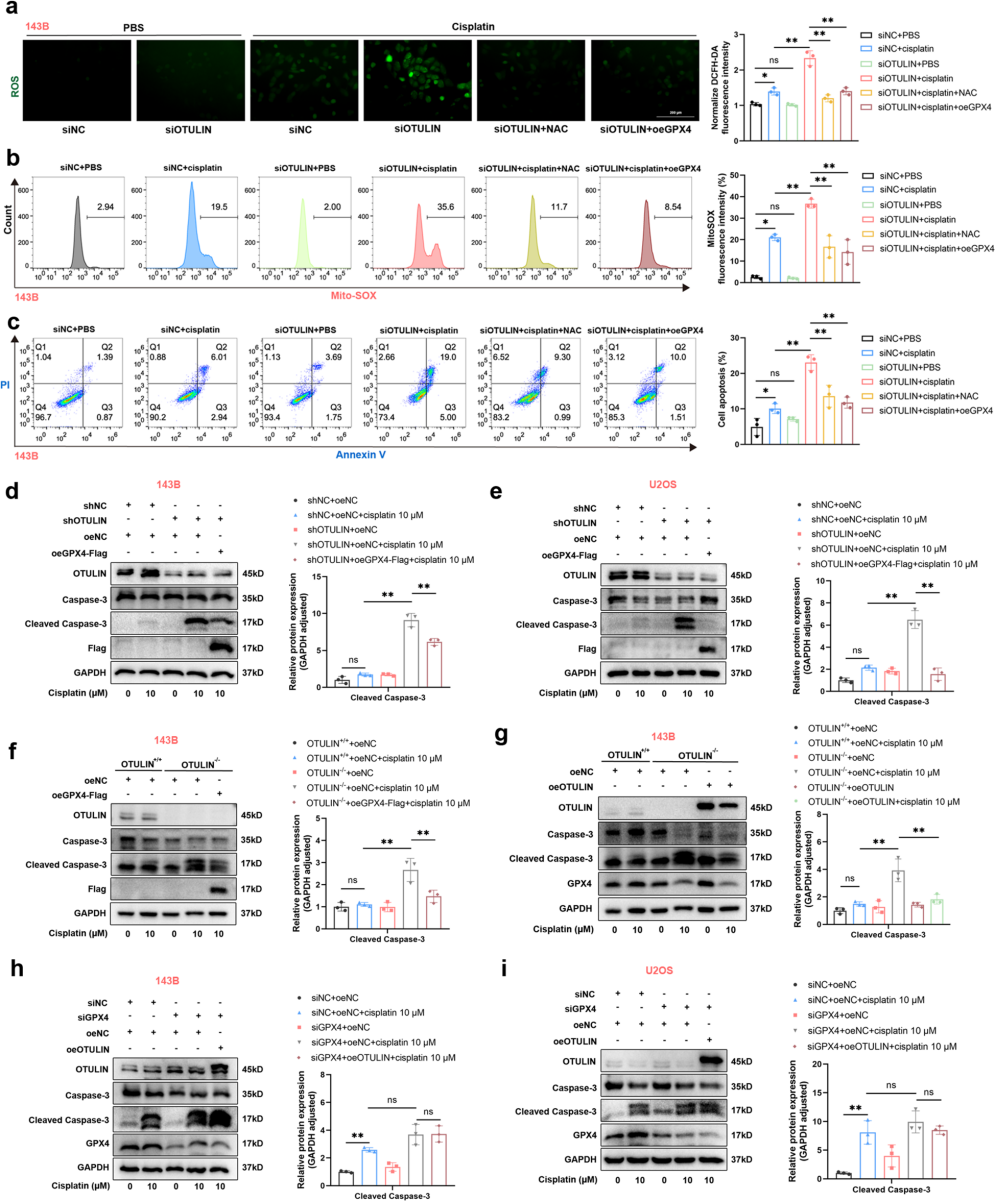
The OTULIN-GPX4 axis conferred resistance to cisplatin in osteosarcoma by blocking the mitochondrial apoptotic pathway. **a-c** OS cells were transfected with siRNA or plasmids for 48 h prior to subsequent interventions. Transfected 143B cells were pretreated with 10 μM NAC for 1 h and then treated with cisplatin (10 μM) for 24 h. **a** Representative images of DCFH-DA staining for ROS and statistical analysis of fluorescence intensity (*n* = 3). **b** Mitochondrial ROS were detected by MitoSOX after treatment, and fluorescence intensity was assessed via flow cytometry (*n* = 3). **c** Apoptosis levels were assessed via flow cytometry (*n* = 3). **d-i** Western blot results and semiquantitative analysis of band density. **d, e** After NC or GPX4 overexpression in lentivirus-transfected (shRNA-NC and shRNA-OTULIN) 143B or U2OS cells for 48 h, cells were treated with cisplatin (10 μM) for 24 h. **f **After transfecting OTULIN-knockout and normal 143B cells with NC or GPX4-overexpression plasmids for 48 h, cells were treated with cisplatin (10 μM) for 24 h. **g** After cells were transfected with NC or OTULIN-overexpression plasmids in OTULIN-knockout and normal 143B cells for 48 h, they were treated with cisplatin (10 μM) for 24 h. **h, i** After transfecting cells with the indicated siRNAs and overexpression plasmids, they were treated with cisplatin (10 μM) for 24 h. The values are expressed as means ± S.D.s. *P < 0.05, **P < 0.01 vs. the corresponding control

Since ROS are known to trigger or promote the mitochondrial apoptotic pathway under cisplatin treatment [14, 15] and GPX4 functions as a resistance molecule to ROS [16], we hypothesized that increased cisplatin-induced apoptosis due to OTULIN depletion in osteosarcoma cells is associated with GPX4 deficiency and increased mitochondrial oxidative stress. To test this hypothesis, we assessed the levels of total ROS (DCFH-DA) and mitochondrial ROS (Mito SOX) in osteosarcoma cells. OTULIN knockdown significantly increased the levels of total and mitochondrial ROS induced by cisplatin, effects that were suppressed by N-acetylcysteine (NAC) and GPX4 overexpression (Fig. 6a-b). Furthermore, the rate of cell apoptosis, which was increased in OTULIN-knockdown cells treated with cisplatin, was reversed by GPX4 overexpression and NAC (Fig. 6c). Cleaved caspase-3 is a crucial protein involved in the mitochondrial apoptosis pathway. [17]. Western blot results demonstrated that GPX4 overexpression significantly reversed the cisplatin-induced increase in the protein level of cleaved caspase 3 in OTULIN-knockdown cells (Fig. 6d, e).

To validate the above results, we constructed a stable OTULIN-knockout 143B cell line via the CRISPR/Cas9 system. The results demonstrated that GPX4 overexpression effectively reversed the cisplatin-induced increase in cleaved caspase-3 protein levels in OTULIN-knockout cells (Fig. 6f). Furthermore, the exogenous overexpression of OTULIN reversed the cisplatin-induced increase in cleaved caspase-3 protein levels in OTULIN-knockout cells (Fig. 6g). However, OTULIN overexpression did not reverse the increase in cleaved caspase-3 levels induced by cisplatin in GPX4-knockdown cells (Fig. 6h, i). Collectively, these findings indicate that GPX4 could be a downstream target of OTULIN that confers resistance to cisplatin in osteosarcoma by blocking the mitochondrial apoptotic pathway.

### OTULIN prevents the proteasomal degradation of GPX4 by reducing its ubiquitin level

**Fig. 7:**
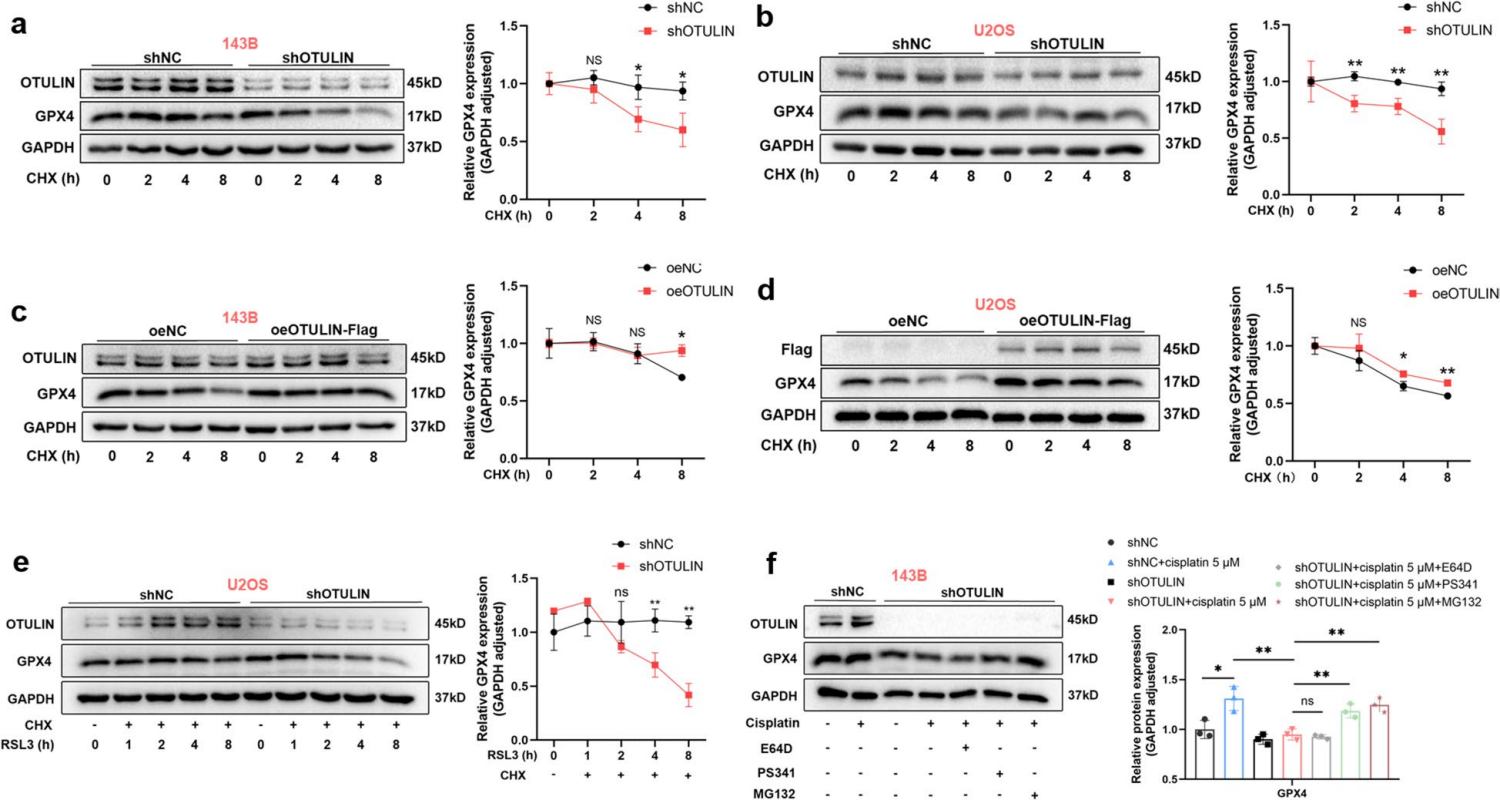
OTULIN regulated the protein stability of GPX4 by preventing its proteasomal degradation. a-e Western blot results and the GPX4 protein decay line chart after treatment (*n* = 3). **a**,** b** Lentivirus-transfected (shRNA-NC and shRNA-OTULIN) 143B or U2OS cells were treated with CHX (100 μM) for the indicated times before harvest. **c**, **d** 143B or U2OS cells were transfected with the overexpression plasmids shown after treatment with CHX (100 μM) for the indicated times before harvesting the cells. **e** Lentivirus-transfected (shRNA-NC and shRNA-OTULIN) 143B cells were treated with CHX (100 μM) for 8 h and RSL3 (0.5 μM) for the indicated times. **f** Lentivirus-transfected (shRNA-NC and shRNA-OTULIN) 143B cells were treated with cisplatin for 24 h, followed by 8 h of treatment with E64D (10 μM), MG132 (10 μM), or PS341 (200 nM). The values are expressed as means ± S.D.s. *P < 0.05, **P < 0.01 vs. the corresponding control

Next, we determined whether OTULIN deficiency sensitizes GPX4 instability by affecting protein synthesis. We found that under conditions in which protein synthesis was inhibited by cycloheximide (CHX), OTULIN deficiency shortened the half-life of the GPX4 protein (Fig. 7a, b), whereas OTULIN overexpression extended the half-life of the GPX4 protein (Fig. 7c, d). Moreover, we used RSL3, a known inducer of ferroptosis, to promote GPX4 degradation and found that the reduction in GPX4 levels in OTULIN-knockdown osteosarcoma cells was further accelerated by treatment with RSL3 together with CHX (Fig. 7e). These results suggest that OTULIN stabilized GPX4 independently of protein synthesis. To determine whether OTULIN affects GPX4 stability associated with proteasomal degradation, osteosarcoma cells were treated with proteasome inhibitors (MG132 and PS341) and a lysosomal inhibitor (E64D) to prevent protein degradation. The results revealed that the decreased protein level of GPX4 induced by OTULIN deficiency could be rescued by PS-341 or MG132 but not by E64D (Fig. 7f). Taken together, these results suggest that OTULIN modulates GPX4 protein stability by preventing proteasomal protein degradation.

**Fig. 8:**
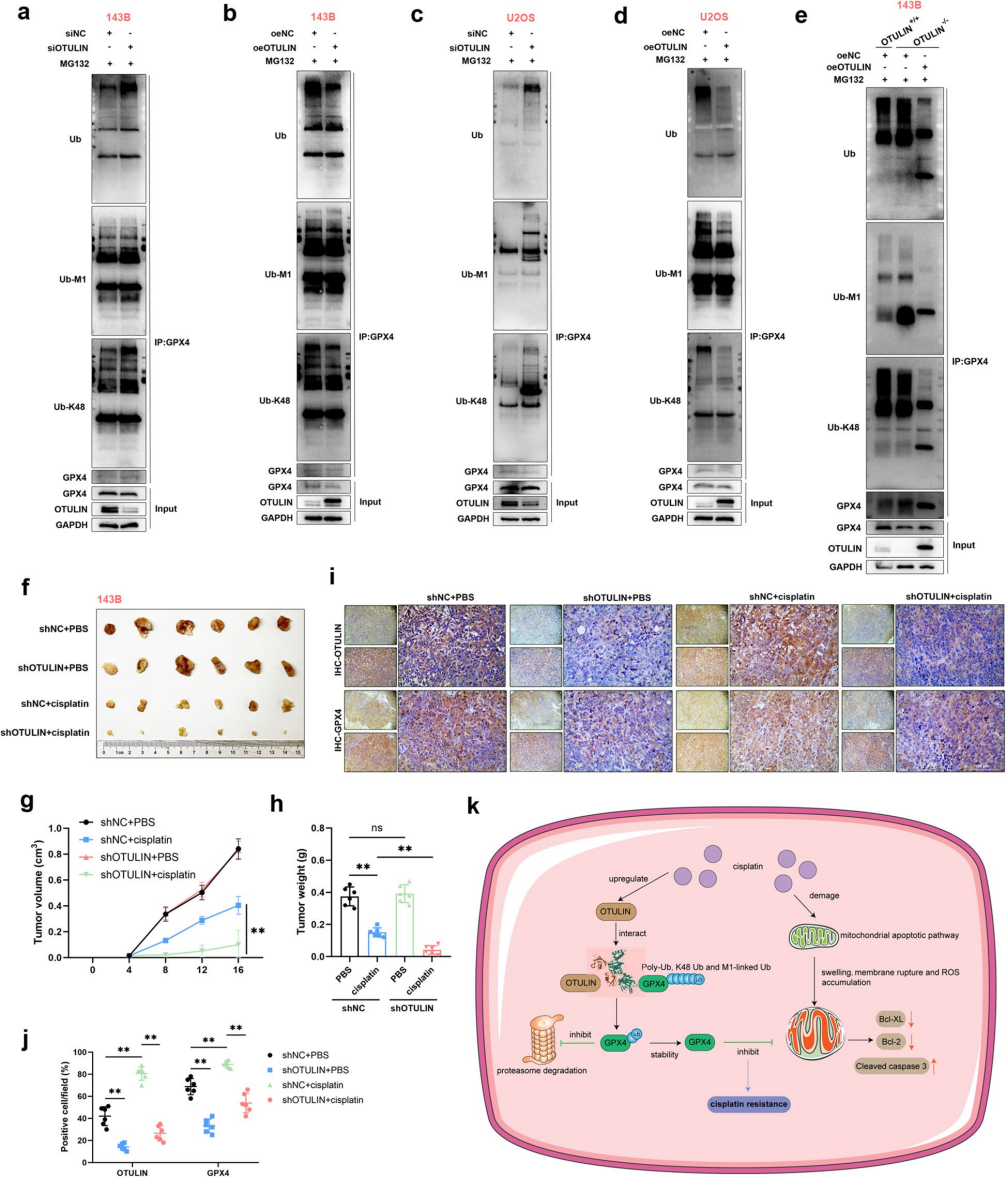
OTULIN regulated the ubiquitin levels of GPX4 and conferred resistance to cisplatin in osteosarcoma. **a-d** To maintain cell number consistency, we transfected 143B or U2OS cells with the indicated siRNAs and overexpression plasmids for 48 h, followed by treatment with MG132 (10 μM) for 6 h before harvesting the cells. The collected lysates were heat denatured for subsequent IP experiments. **e** After transfecting an overexpression plasmid into OTULIN knockout or normal 143B cells for 48 h, the cells were treated with MG132 (10 μM) for 6 h. **f** Images of 143B-derived tumors in nude mice. **g**,** h** Tumor volume and tumor weight were measured and analyzed. **i** Representative IHC images of OTULIN and GPX4. **j** Quantitative analysis of OTULIN- and GPX4-positive cells (*n* = 6). **k** Graphical abstract. The values are expressed as means ± S.D.s. *P < 0.05, **P < 0.01 vs. the corresponding control

Since OTULIN deficiency sensitized GPX4 to degradation, we next characterized how OTULIN affects the ubiquitination status of GPX4. To ensure that the altered ubiquitination was of GPX4 itself and not its interactors, total lysates were boiled to denature the protein to break noncovalent bonds prior to antibody immunoprecipitation. We found that OTULIN knockdown significantly increased the ubiquitin levels of the poly-Ub, K48 and M1-linked chains of GPX4 (Fig. 8a, c); conversely, OTULIN overexpression decreased the ubiquitin levels of the poly-Ub, K48 and M1-linked chains (Fig. 8b, d). To verify the accuracy of these results, we overexpressed exogenous OTULIN in the 143B OTULIN-knockout cell line and found that exogenous OTULIN expression significantly inhibited the level of ubiquitination of polyubiquitin, the K48 chain, and the M1 chain on GPX4 (Fig. 8e). Collectively, these results indicate that OTULIN prevents the proteasomal degradation of GPX4 by reducing its ubiquitin level.

### OTULIN confers resistance to cisplatin in osteosarcoma

We then investigated in vivo whether OTULIN is associated with cisplatin resistance in osteosarcoma cells. Nude mice with xenografts of shRNA-NC and shRNA-OTULIN 143B cells were treated with PBS or cisplatin. We found that the OTULIN-normal and OTULIN-knockdown groups had similar proliferation rates; however, OTULIN-knockdown 143B cells exhibited greater sensitivity to cisplatin than OTULIN-normal cells did (Fig. 8f–h). This finding is consistent with the results of previous in vitro experiments. The results demonstrated that altering OTULIN expression alone did not alter the proliferative capacity of osteosarcoma cells but that OTULIN deficiency significantly reduced the resistance of osteosarcoma to cisplatin. The immunohistochemical results revealed that cisplatin indeed upregulated the expression of OTULIN and GPX4; concomitantly, OTULIN knockdown significantly inhibited the cisplatin-induced increase in GPX4 expression in vivo (Fig. 8i, j). These observations demonstrate that OTULIN confers cisplatin resistance in vivo.

## Discussion

Cisplatin, a chemotherapeutic agent commonly used to treat OS, triggers the formation of incorrect cross-links by binding to DNA, leading to DNA damage and initiating cell death [18, 19]. However, an increasing number of studies have reported that signals that promote cell death may not be fully activated despite the presence of DNA damage, in part because the signaling molecules that promote cell death are mutated or their expression is downregulated and, in part, because the expression of signaling molecules that prevent cell death is upregulated [20, 21]. In the present study, we found that OTULIN can act as a key molecule in promoting cisplatin resistance; this cisplatin-dependent upregulation of OTULIN expression markedly decreased the sensitivity of osteosarcoma cells to cisplatin-induced cell death.

OTULIN, to date, is the only mammalian DUB that exclusively hydrolyzes Met1-linked Ub chains [22]. Different groups have reported that OTULIN deficiency can lead to severe autoinflammatory diseases called OTULIN-related autoinflammatory syndrome (ORAS, also termed otulipenia) [23, 24]. According to recent reports, the types of cell death regulated by OTULIN include apoptosis [5, 25] and necroptosis [26]. Indeed, our data also revealed that OTULIN deficiency increases the sensitivity of osteosarcoma cells to apoptosis and necroptosis induced by cisplatin. The novel results revealed that the proapoptotic effect of OTULIN deficiency is importantly linked to its regulation of GPX4. Interestingly, our results revealed that GPX4 overexpression significantly reversed the proapoptotic effects of OTULIN knockdown, whereas OTULIN overexpression did not reverse the proapoptotic effects of GPX4 knockdown. Previous studies have also shown that cisplatin promotes GPX4 expression, although the mechanism was not fully elucidated in their study [10]. In our study, we found that the high expression of GPX4 after cisplatin treatment was highly dependent on OTULIN. Therefore, we propose that GPX4 is a critical target of OTULIN for resistance to cisplatin-induced apoptosis in OS.

Emerging evidence suggests that nonmutational drug resistance mechanisms play a key role in allowing ‘persister’ cancer cells to survive [27, 28]. Our results suggest that the OTULIN-GPX4 axis plays an essential nonmutational role in cisplatin resistance. To date, the majority of studies examining the mechanisms by which OTULIN regulates cell death have focused on its impact on RIPA [29], RIPK1 [30], NF-κB and TNF signaling [5, 31, 32]. However, few studies have noted the involvement of the antiapoptotic effects of OTULIN in association with oxidative stress. Many studies have reported that ROS play an important role in triggering apoptosis in cancer cells through the mitochondrial pathway [33–35]. Numerous previous studies have confirmed that cisplatin triggers ROS overproduction, an important cytotoxic effect [9, 36, 37]. Our results revealed that OTULIN deficiency significantly promoted cisplatin-induced total and mitochondrial ROS production, and as expected, these effects were reversed by GPX4 overexpression or NAC. The JC-1 and electron microscopy results also indicated that OTULIN deficiency exacerbated cisplatin-induced mitochondrial damage. Previous studies have indicated that GPX4, an antiapoptotic factor, primarily utilizes GSH as a cofactor to resist lipid peroxidation, thereby protecting membrane integrity [13, 38]. Mechanistically, high GPX4 expression protects cells from apoptosis mediated by the mitochondrial pathway by blocking the release of CYCS from mitochondria, inactivating caspase3, and inhibiting the production of hydrogen peroxide [16]. An increasing number of studies have reported a close association between high GPX4 expression and chemoresistance [39]. Highly mesenchymal, drug-resistant cancer cells depend on the lipid hydroperoxidase GPX4 to survive [40]. More interestingly, the loss of GPX4 function results in selective ferroptosis in vitro and prevents tumor recurrence in vivo since ‘persister’ cells acquire a dependency on GPX4 [41]. Recent studies have shown that GPX4, an oxidoreductase that scavenges lipid peroxidation, increases cellular resistance to apoptosis and ferroptosis [16, 42]. Our results also revealed that GPX4 deficiency promoted cisplatin-induced apoptosis but did not significantly promote ferroptosis, which seems to be inconsistent with the findings of a previous study [10]. In addition, our results also revealed that cisplatin did not significantly increase lipid peroxidation in osteosarcoma cells, with or without OTULIN knockdown. On the basis of these reports and our results, we suggest that the OTULIN-GPX4 axis inhibits cisplatin-induced mitochondrial damage-dependent apoptosis but not ferroptosis.

Our results indicated that high GPX4 expression after cisplatin intervention was significantly regulated by OTULIN, as OTULIN deubiquitylated GPX4 to reduce its proteasomal degradation. Similar functions of OTULIN have been reported in previous studies, where the absence of OTULIN in certain cells, such as fibroblasts, led to the proteasomal degradation of LUBAC components [22, 23, 43]. A recent study revealed that linear ubiquitin, which is regulated by LUBAC, contributes to GPX4 protein homeostasis [44]. However, we provide novel evidence that OTULIN deficiency promotes the ubiquitination of GPX4, including linear ubiquitin and K48, in osteosarcoma cells, which promotes the proteasomal degradation of GPX4. In fact, in a previous study, OTULIN deficiency reduced the protein levels of GPX4, but how OTULIN exerts this effect was not investigated [44]. Our results revealed that GPX4 linear ubiquitination, which was upregulated by OTULIN insufficiency, did not promote GPX4 protein stabilization; however, the concomitant increase in K48-linked ubiquitin on GPX4 tended to promote the proteasomal degradation of GPX4. Owing to the specificity of the linear deubiquitination of OTULIN, it remains unclear how OTULIN deficiency leads to an increase in other ubiquitin chains on GPX4, such as K48 and poly-Ub, in addition to linear ubiquitination. Nevertheless, our work revealed a role for OTULIN in regulating GPX4 protein stability and confirmed that the antiapoptotic effects associated with OTULIN and GPX4 are closely related to cisplatin resistance.

Notably, this study is not without limitations. First, we did not clarify how cisplatin stimulated high OTULIN expression, and we believe that this mechanism still deserves in-depth investigation. Second, the connection and interaction between the levels of different types of ubiquitin on GPX4 remain unclear, and the crosstalk and interactions between different types of ubiquitin deserve further investigation.

## Conclusion

This study provides insight into the mechanism of OTULIN in the chemoresistance of OS and reveals that it inhibits cisplatin-induced apoptosis by modulating the stability of the GPX4 protein, thereby conferring resistance to chemotherapeutic agents in OS cells. Specifically, OTULIN was markedly elevated following chemotherapy, particularly in response to cisplatin treatment, but not to doxorubicin or methotrexate. OTULIN overexpression maintained the stability of the GPX4 protein by reducing the level of ubiquitination of GPX4, which in turn inhibited cisplatin-induced apoptosis without affecting the ferroptosis process. These findings provide new insights into OS chemoresistance and suggest that the OTULIN-GPX4 axis may be a promising therapeutic target for improving chemotherapeutic outcomes in OS patients.

## Supplementary Information

Supplementary Material 1

Supplementary Material 2

## Data Availability

The datasets supporting the conclusions of this article are included within the article and its additional files.

## Abbreviations

CHX: Cycloheximide
CDTX: Cell line derived tumor xenograft
CCK 8: Cell counting kit 8
DOX: Doxorubicin
IHC: Immunohistochemistry
IF: Immunofluorescence
IP: Immunoprecipitation
KO: Knockout
LC–MS: Liquid chromatography-mass spectrometry
MTX: Methotrexate
NAC: N-Acetylcysteine
OS: Osteosarcoma
OTULIN: OTU deubiquitinase with linear linkage specificity
ORAS: OTULIN-related auto-inflammatory syndrome
ROS: Reactive oxygen species
siRNA: Small interfering RNA
SPF: Specific pathogen-free
TCGA: The Cancer Genome Atlas Program
TEM: Transmission electron microscopy
KEGG: Kyoto Encyclopedia of the Genome
