# Supplementary material for: OTULIN confers cisplatin resistance in osteosarcoma by mediating GPX4 protein homeostasis to evade the mitochondrial apoptotic pathway
Source: J Exp Clin Cancer Res. 2024 Dec 26;43:330. doi: 10.1186/s13046-024-03249-8 (PMC11670407; doi:10.1186/s13046-024-03249-8)
Supplement: Supplementary file 1 — Supplementary Material 1 [file 13046_2024_3249_MOESM1_ESM.docx]

**Table S1. Patients’ tumor information.**

| Sample ID | Age | Gender | Location of the tumor | Neoadjuvant chemotherapy regimen |
| --- | --- | --- | --- | --- |
| 1 | 17 | male | femur | Cisplatin, doxorubicin and methotrexate |
| 2 | 20 | male | tibia | Cisplatin, doxorubicin and methotrexate |
| 3 | 15 | male | femur | Cisplatin, doxorubicin and methotrexate |
| 4 | 15 | Female | tibia | Cisplatin, doxorubicin and methotrexate |
| 5 | 14 | Female | femur | Cisplatin, doxorubicin and methotrexate |
| 6 | 12 | Female | femur | Cisplatin, doxorubicin and methotrexate |
|  |  |  |  |  |

**Table** **S2. Primers.**

| Primers. | Sequences |
| --- | --- |
| SLC7A11（F） | TCCTGCTTTGGCTCCATGAACG |
| SLC7A11（R） | AGAGGAGTGTGCTTGCGGACAT |
| GPX4 (F) | ACAAGAACGGCTGCGTGGTGAA |
| GPX4 (R) | GCCACACACTTGTGGAGCTAGA |
| ACSL4 (F) | GCTATCTCCTCAGACACACCGA |
| ACSL4 (R) | AGGTGCTCCAACTCTGCCAGTA |
| OTULIN (F) | GTACCGTGCTGCAGATGAAA |
| OTULIN (R) | GGCCCTCAGTGCACAGTAAT |

**Table** **S3. siRNA and sgRNA**

| siRNA | Sequences |
| --- | --- |
| siOTULIN-1 | GCUUGUGAUGAACUAUUCA |
| siOTULIN-1 | UGAAUAGUUCAUCACAAGC |
| siOTULIN-2 | GCUCAUGCUGUUACCAGAA |
| siOTULIN-2 | UUCUGGUAACAGCAUGAGC |
| siOTULIN-3 | GCAUCAGAACCGAGAUUAA |
| siOTULIN-3 | UUAAUCUCGGUUCUGAUGC |
| siGPX4-1 | GGAAGUGGAUGAAGAUCCA |
| siGPX4-1 | UGGAUCUUCAUCCACUUCC |
| OTULIN-sgRNA-1# | TAAGCAGCTGTATAATAGGA |
| OTULIN-sgRNA-2# | AGAGGGGCATCAGGTATGTT |

**
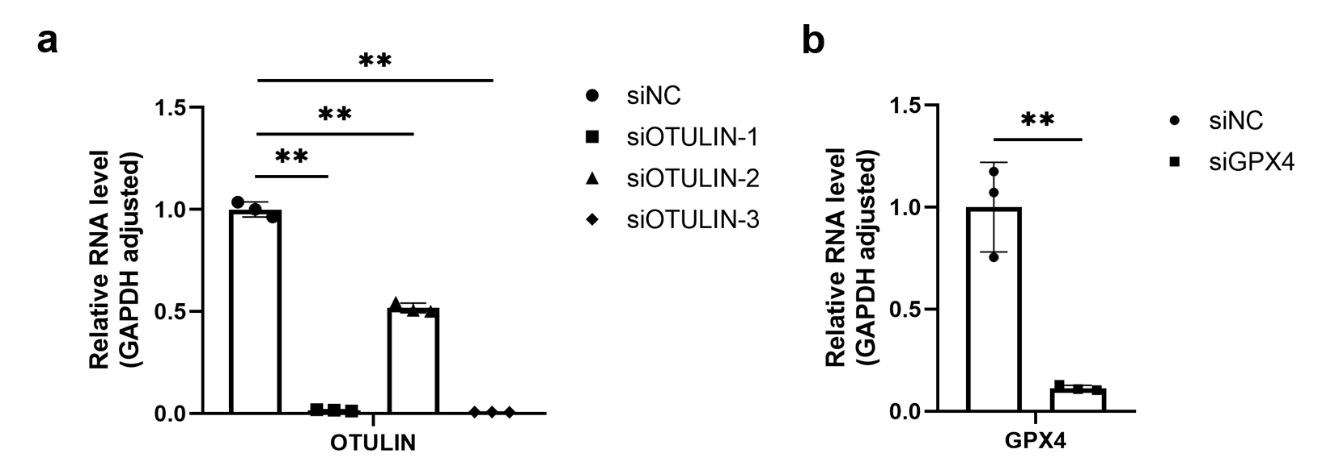
**

**sFig. 1 gene interference efficiency.**
